# Marine ω-3 PUFA Supplementation Enhances FFAR4 Activation and Reduces Inflammatory Markers in PBMC of Subjects with Obesity: A Randomized Controlled Trial (EPICO)

**DOI:** 10.3390/nu17233630

**Published:** 2025-11-21

**Authors:** Samantha Desireé Reyes-Pérez, Diego Cambron-Mora, Joel Torres-Vanegas, Karla Lizette Mojica-Zamudio, Carolina Elizabeth Olaez-Ramos, Ramon Gerardo Lauriano-Rivera, Juan J. Rivera-Valdés, Nathaly Torres-Castillo, Evelyn Valencia-Sosa, Roberto Rodriguez-Echevarria, Erika Martinez-Lopez

**Affiliations:** 1 Instituto de Nutrigenética y Nutrigenómica Traslacional, Centro Universitario de Ciencias de la Salud, Universidad de Guadalajara, Guadalajara 44340, Jalisco, Mexico; samantha.reyes@academicos.udg.mx (S.D.R.-P.); diegocambron21@gmail.com (D.C.-M.); joel.torres3337@alumnos.udg.mx (J.T.-V.);; 2 Doctorado en Ciencias en Biologia Molecular en Medicina, Centro Universitario de Ciencias de la Salud, Universidad de Guadalajara, Guadalajara 44340, Jalisco, Mexico; 3 Doctorado en Ciencias de la Nutrición Traslacional, Centro Universitario de Ciencias de la Salud, Universidad de Guadalajara, Guadalajara 44100, Jalisco, Mexico; 4 Licenciatura de Nutrición, Centro Universitario de Ciencias de la Salud, Universidad de Guadalajara, Guadalajara 44340, Jalisco, Mexico; 5 School of Medicine and Health Sciences, Tecnologico de Monterrey, General Ramon Corona Ave., 2514, Zapopan 45138, Jalisco, Mexico

**Keywords:** FFAR4, GPR120, ω-3 PUFA, chronic inflammation, obesity, marine oil, clinical trial

## Abstract

**Background:** It is widely accepted that low-grade chronic inflammation in obesity worsens the metabolic state and threatens patients’ lives in a long-term manner. In fact, diet therapy is the first-line treatment in which relevant nutrients such as the omega-3 polyunsaturated fatty acids (ω-3 PUFA) must be adequately consumed to counteract the established inflammation. In particular, eicosapentaenoic acid (EPA) and docosahexaenoic acid (DHA) have been identified as agonists of cellular receptors, including the free fatty acid receptor 4 (FFAR4), which regulates anti-inflammatory pathways associated with enhanced insulin sensitivity. However, the expression and activation of this receptor in peripheral blood mononuclear cells (PBMC) remains poorly investigated in humans. **Objective:** This study aimed to evaluate the effect of a diet supplemented with marine ω-3 PUFA on FFAR4 receptor activation and inflammatory markers in peripheral blood mononuclear cells in subjects with obesity. **Methodology:** A double-blind, randomized clinical trial (NCT05068557) was conducted over two months (eight weeks) in 55 obese individuals (aged 25–59 years). Participants were randomly assigned to one of two groups: an active placebo group (1.6 g/day of alpha-linolenic acid) or a marine ω-3 group (1080 mg of EPA and 720 mg of DHA). Both groups followed a dietary regimen with progressive calorie restriction (−200 kcal/day during weeks 0–4 and −400 kcal/day during weeks 4–8) in addition to supplementation. **Results:** Following the intervention, both groups showed significant improvements in body composition and biochemical parameters. Supplementation with EPA and DHA enhanced FFAR4 activation at the end of the intervention. Moreover, there was a reduction in the expression of *JNK* and *IKKβ* genes, as well as in serum levels of TNF-α, IL-6, and IL-18. In contrast, IL-10 levels increased significantly both within and between the groups. **Conclusions:** Marine ω-3 PUFA supplementation, in the context of a dietary intervention, promotes FFAR4 activation, thereby contributing to the modulation of the inflammatory response in human PBMC.

## 1. Introduction

Obesity represents a major and largely unresolved public health issue [1]. In fact, it has tripled since 1975, from 4% to 13% in 2016 [2]. Thus, it is remarkable that despite the multiple treatment strategies at disposal, such as nutritional counseling, psychological therapy, physical activity prescription, drug therapy, and bariatric surgery, obesity remains widely unmanageable in the epidemiological scale [3]. Thus, these alternatives reinforce the idea that innovative and structured strategies should be created to treat and most importantly, prevent this condition and its associated comorbidities [4].

Among the most common disorders derived from obesity are low-grade chronic inflammation and metabolic alterations [5]. It is widely acknowledged that a high percentage of body fat mass is the cornerstone of a progressive metabolic dysfunction [6]. In fact, the underlying molecular mechanisms explaining these alterations are described to be driven by a low-grade chronic inflammation involving immune cell infiltration of adipose tissue [7]. One of the reasons for the onset of this phenomenon is the hypertrophic state, promoted by a sustained positive energy balance over time. During this expansion, oxidative stress is generated, followed by cellular hypoxia leading to cell death, which is a key feature leading to the release of fatty acids, chemotactic mediators, and the infiltration of immune cells. In fact, this worsens the condition by promoting the polarization of M2 macrophages into the M1 phenotype which increases the production of proinflammatory cytokines such as tumor necrosis factor alpha (TNFα), interleukin 1β (IL-1β), and interleukin 6 (IL-6) [8], altering the physiological regulation of lipid metabolism and insulin signaling through a crosstalk mechanism of relevant signaling pathways in the adipocytes [9].

As previously mentioned, diet therapy has long been recognized as the first-line intervention for preventing and reducing low-grade chronic inflammation. Among the most extensively studied nutrients in this context are omega-3 polyunsaturated fatty acids (ω-3 PUFA), particularly eicosapentaenoic acid (EPA) and docosahexaenoic acid (DHA) [10]. Recent research has focused on molecules involved in the activation of anti-inflammatory pathways, representing a significant advancement in obesity treatment. One such molecule is the FFAR4 receptor (Free Fatty Acid Receptor 4), also known as GPR120 (G protein-coupled receptor 120), which is primarily expressed in the colon, adipose tissue, and liver. This transmembrane receptor plays a key role in multiple signaling pathways that regulate cellular functions. FFAR4 exhibits tissue-specific activity, enhances insulin sensitivity, and modulates inflammatory responses through its ligands, the ω-3 PUFA EPA and DHA, which are well-documented for their anti-inflammatory properties [11,12].

Mechanistically, FFAR4 activation triggers two principal signaling routes: a Gαq-dependent pathway that stimulates phospholipase C (PLC), leading to downstream activation of the phosphatidylinositol 3-kinase (PI3K) cascade and increased glucose uptake via glucose transporter type 4 (GLUT4) translocation; and a β-arrestin-2–dependent pathway responsible for the receptor’s anti-inflammatory effects. The latter involves internalization of the FFAR4/β-arrestin-2 complex and subsequent sequestration of TAK1-binding protein 1 (TAB1), which prevents activation of the proinflammatory transforming growth factor β–activated kinase 1 (TAK1) and downstream phosphorylation of inhibitor of nuclear factor kappa B kinase β (IKKβ), mitogen-activated protein kinase kinase 4 (MKK4), and c-Jun N-terminal kinase (JNK), ultimately reducing nuclear factor kappa-light-chain enhancer of activated B cells (NF-κB) nuclear translocation and cytokine transcription. Through these mechanisms, FFAR4 activation contributes to improved insulin sensitivity and attenuation of chronic inflammation associated with obesity [13,14].

Studies in vivo and in vitro models have demonstrated that FFAR4 activation through ω-3 supplementation reduces immune cell infiltration, promotes glucose uptake, and improves insulin sensitivity [15]. Moreover, FFAR4 is expressed in peripheral blood mononuclear cells (PBMCs), particularly in monocytes, making them a relevant and accessible model to evaluate diet-induced modulation of inflammatory signaling in humans. Given this evidence, the present trial aimed to assess whether marine ω-3 PUFA supplementation enhances FFAR4 activation in PBMCs and thereby contributes to the reduction in inflammatory markers in individuals with obesity. A simplified mechanistic diagram of FFAR4 signaling through Gαq- and β-arrestin-2–dependent pathways is provided to illustrate the proposed molecular mechanisms.

## 2. Subjects and Methods

### 2.1. Study Design and Settings

**Figure 1:**
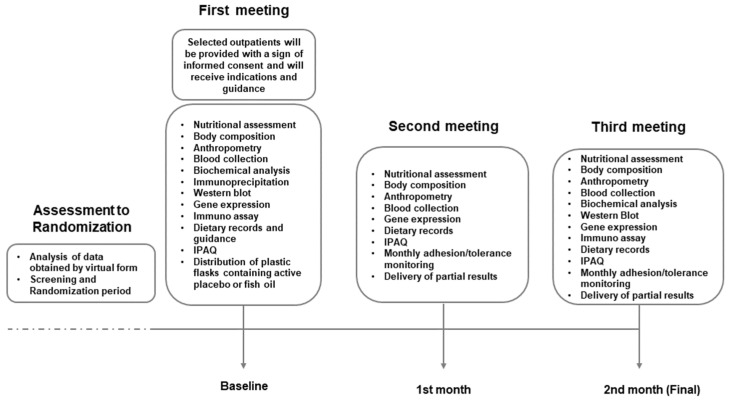
Experimental interventions scheme and detailed subject flow.

This study was designed to strategically evaluate the anti-inflammatory and pro-resolving markers derived from a consistent consumption of marine ω-3 PUFA in a non-invasive approach through PBMC genomics with serum markers from the nutrigenomics standpoint. This study is a single-centered, double-blinded, randomized active placebo-controlled intervention trial and it is aligned with the Standard Protocol Items: Recommendations for Interventional Trials (SPIRIT) checklist. The study was approved by the Ethics, Research, and Biosafety Committee from CUCS, University of Guadalajara (CI03221) and registered at ClinicalTrials.gov (NCT05068557). Importantly, all included subjects were thoroughly informed and provided with an informed consent form to be signed. The duration of the intervention was two months (8 weeks), during which participants attended a total of three scheduled visits to the facility (Figure 1).

### 2.2. Eligibility of Participants

Basic eligibility criteria considered adults with an age range of 25–59 years with obesity (Body Mass Index (BMI) 30–39.9 kg/m^2^) or with abdominal obesity (waist circumference > 88 cm for women and >102 cm for men) without major health complications at the time of recruitment. The cohort consisted of outpatients who were willing to attend the Instituto de Nutrigenética y Nutrigenómica Traslacional (INNUGET). Furthermore, the non-inclusion criteria also considered any previous occurrence of chronic infections or official diagnosis of conditions such as cardiovascular, metabolic, endocrine, neoplastic or autoimmune diseases. Also, pregnant women, breastfeeding mothers, or subjects with a record of alcohol abuse were not included. Additionally, patients who have been under any type of surgery involving the gastrointestinal tract or gallbladder, or unable to undergo the standardized anthropometric evaluation due to amputated limbs, were not considered eligible. Furthermore, individuals consuming anti-inflammatory, lipid-lowering, or hypoglycemic drugs or who had consumed nutritional supplements containing essential fatty acids within the last three months prior to the study were not eligible. Lastly, individuals who have experienced a weight loss exceeding 10% of their body weight within the past three months were not enrolled in the intervention.

Importantly, subjects who were diagnosed with any of the previously mentioned diseases during the study, notice of pregnancy or subjects experiencing discomfort associated with possible side effects opting to withdraw were excluded from the analyses.

### 2.3. Calculation of Sample Size

We determined the sample size using the G*Power 3.1.9.7 software. Considering the Omega-PAD II trial [16] outcomes in which resolvin E3 (RvE3) was the only specialized pro-resolving mediator (SPM) with a statistically significant increase in both intergroup and intragroup analysis (*p* = 0.04 and *p* = 0.04). It was also the most abundant in the treated group, reflecting a marked and traceable variation due to active ω-3 PUFA supplementation. Based on an effect size of *d* = 0.91 between RvE3 in the placebo group (58 ± 82 pg/mL) and the treated group (187 ± 199 pg/mL), and to minimize type II errors, with a type I error set at α = 0.05 and a power (1 − β) of 80%, a total 20 individuals in each group were required. Finally, based on the previous result and considering potential dropout rates of up to 30% commonly reported in obesity-related randomized clinical trials (RCTs) [17], the final sample size was increased to 26 participants per group.

### 2.4. Recruitment

The study spanned from September 2021 to December 2023. There was a total of 332 interested individuals from the Guadalajara metropolitan area in Mexico who were assessed for eligibility through a Google Forms survey posted on various social media platforms. The diffusion of the study was carried out under the name “EPICO” (Estudio para la Prorresolución de la Inflamación Crónica en Obesidad) which is an acronym derived from Spanish meaning “Study for the Pro-resolution of Chronic Inflammation in Obesity”. Of note: for the purpose of this publication, the acronym was maintained despite minor changes in the wording but maintaining the general meaning.

For each participant, there were a maximum of 3 attempts to reach after the first contact; a total of 80 participants were recruited. Each participant was provided with a copy of the informed consent that was required to sign upon agreement. The procedures followed were conducted according to the Helsinki Declaration (2013) [18].

### 2.5. Randomization

The randomization of the groups was carried out by the permuted blocks technique [19,20]. This method provides a way to randomly allocate one patient to a treatment group within a set of study participants, which is called a block. Each block had a specific number of randomly ordered treatment assignments. Block sizes are multiples of the number of treatments and take the allocation ratio into account. Furthermore, this type of randomization maintains a proper balance between treatment groups. That means generating the randomization code according to the order of selection of each block. In this study, blocks of 8 participants were used to maintain a 1:1 allocation ratio.

In this study, conducting a trial with two intervention groups, it was considered that a block of two elements would be predictable, so it was ideal to use ten blocks with a size of eight elements to ensure the proportion. Subsequently, a list of the possible permutations of two elements taken four by four was made. The blocks were randomly ordered from one to eight and the participants were successively assigned to the option that corresponds to them, filling the blocks randomly. In this way, every four assigned participants equalized the number of participants in each intervention group.

### 2.6. Intervention

On every visit, the patients received a nutritional counseling session guided by a trained dietitian, and they were scheduled for monthly appointments for a two-month period. The first visit comprised a complete evaluation including the fill of the clinical history of the patient: sociodemographic data, alcohol consumption, smoking, prescribed medications, a bioelectrical impedance procedure for the assessment of body composition and anthropometric profiles, a 24 h dietary recall, the application of the International Physical Activity Questionnaire (IPAQ) to estimate the level of physical activity, and collection of blood samples in the fasting state for at least 8–10 h.

In this first visit patients were randomized to receive either 1.8 g ω-3 PUFA (1080 mg EPA + 720 mg DHA; purchased from General Nutrition Center Mexico (GNC^®^, Pittsburgh, PA, USA) Double Strength Fish Oil) or a matching active placebo (chia and flaxseed oil containing 1.6 g of alpha-linolenic acid (ALA)) purchased from Biocaps Mexico^®^, San Miguel Ajusco, Mexico. The total dose was divided into 3 capsules to be taken with meals three times a day.

The second part of the intervention comprised a semi-personalized diet with a mild calorie restriction progressing from 200 to 600 kilocalories at every meeting according to the energy expenditure formula from Mifflin-St Jeor. All subjects were guided to their assigned calorie prescription based on a calorie chart consistent with the food servings including those in the list of food groups (equivalents). The calorie chart was segmented from 1200 to 2400 calories with a 5:1 ω-6: ω-3 ratio previously calculated by Nutritionist Pro^TM^ version 8.1 software (Axxya Systems, Woodinville, WA, USA). The distribution of macronutrients was 55% carbohydrates, 25% lipids (Saturated Fatty Acids (SFA) < 7%, Monounsaturated Fatty Acids (MUFA) ≥ 20% and PUFA ≥ 10%), proteins 20% and sugars < 5% of the total kilocalories. The meal plans consisted of a graphic and descriptive recipe book containing 20 days of healthy options for breakfast, snacks, lunch, and dinner in accordance with the guidelines of the clinical practice guide for dietary intervention in patients with obesity, as well as the guidelines of NOM-043-SSA2-2012 “Health Basic Services, Promotion and Education for Health in Food Matter” [21]. In addition to being broadly based on the nutritional distribution recommended by NOM-037-SSA2-2012, for the prevention, treatment, and control of dyslipidemias [22].

The nutritionist explained to the participant in detail the dietary intervention. Participants were instructed to follow the dietary instructions until the next appointment. Finally, in order to promote an optimal level of adherence to the meal plan, participants were provided with a digital kitchen scale and a full measuring cup set.

### 2.7. Blinding

All individuals involved in the recruitment were unaware of the treatment randomization throughout the trial, including but not limited to: PhD students, researchers, dietitians, and assessors. To enforce blinding, capsules with similar sizes but moderately different in color (due to the source, animal versus vegetable) were deposited in numbered and indistinguishable containers. These containers were prepared by a third-party research team from the campus University who were not involved in the study other than supporting our project with the capsule packing in the containers, labeling included a unique code, as well as the investigators’ contact information and supplementation instructions. This approach guaranteed that both participants and investigators remained completely unaware of the treatment identities. In the event of adverse effects, dietitians were given the option to reveal the administered treatment to participants if they considered it necessary.

### 2.8. Nutritional Content

The fatty acid content of the supplement reported by the commercial products is presented in Tables S1 and S2.

The complete fatty acid profiles of both the treatment and active placebo are shown in Tables S3 and S4. These data were obtained through gas chromatography analysis conducted at an external laboratory.

### 2.9. Anthropometric Assessment

Anthropometric evaluation was carried out by a trained nutritionist according to ISAK (The International Society for the Advancement of Kinanthropometry) [23] and NHANES (National Health and Nutrition Examination Survey) [24] standardized guidelines and consisted of two phases. First, arm [25], neck [26], waist [27], abdominal [28] and hip [29] circumferences were measured with a Lufkin Rosscraft metal tape measure model W606, with a scale of 0 to 200 cm and precision of 0.1 cm.

Subsequently, the evaluation of body composition (percentage of body fat and weight) was carried out with the InBody 570 electric bioimpedance scale. These data were evaluated and interpreted following the respective standardized guidelines and reference cut-off points.

Data were assessed at baseline and post-intervention following respective standardized guidelines and cut-offs.

### 2.10. Assessment of Dietary and Supplement Consumption

Dietary intake data were obtained using a 24 h dietary recall in the first meeting and a 3-day food record in a diary for the follow-up. The latter was required to be filled on two non-consecutive days during the week and one on the weekend. Participants received a guide to correctly fill out the food diary with specifications for each section and resolution of possible questions that arose from using the format.

The consumption of kilocalories, macronutrients (carbohydrates, proteins, lipids), ω-3 PUFA and ω-6 PUFA were the main elements evaluated. These data were analyzed using the Nutritionist pro^TM^ version 8.1 software (Axxya Systems, Woodinville, WA, USA).

As for the supplementation, patients were required to register their supplement consumption in a paper form that was provided on every visit. Additionally, all subjects were asked to return the supplement bottle with any remaining capsules to every following meeting with the aim of double-checking and comparing the count of capsules consumed with the record.

The proposed strategy for assessing comprehensive adherence to the intervention involved evaluating its main components: consumption of capsules and the dietary plan, where the latter was subdivided into two specific areas of interest for the study: kilocalories and the ratio of consumed polyunsaturated ω-6 and ω-3 PUFA.

To establish the cutoff points for the dietary evaluation, kilocalories were considered with a ±10% surplus or deficit as percentages of adequacy. Regarding the consumption ratio of ω-6 and ω-3 PUFA, “low” adherence values were based on those reported by Campos and colleagues [30], which show the average consumption of adults with obesity in the western region of the country to be 12:1. “Optimal” adherence was defined as the recommended average ratio of ≤5:1 [31], as recommended by the recipes included in the cookbook.

The other component of the evaluation was the supplementation, where adherence was assessed by quantifying the consumption of capsules. This quantification was obtained by tracking capsule consumption and was weighted based on the number of capsules not taken. The adherence ranges from “optimal” consumption, where the subject did not consume up to 8 capsules, to “low” adherence, where the subject did not consume 27 capsules or more within a month.

### 2.11. Physical Activity

The IPAQ (International Physical Activity Questionnaire) was used to assess physical activity levels at baseline and after the intervention. The validated IPAQ aims to determine the type of physical activity that the participants perform in their daily lives. The questions refer to the time they spend being active or sedentary in the last seven days [32]. Upon first meeting, participants were requested not to significantly modify their physical activity patterns; nevertheless, if this had occurred, it was evaluated and considered in the analyses.

### 2.12. Monitoring and Retention of Participants

Also, the patients could contact their assigned dietitian for the resolution of questions and doubts through WhatsApp^®^ and schedule a nutritional education session via Zoom^®^ in between their visits. In addition, we created social media accounts (Facebook^®^ and Instagram^®^) with the purpose of sharing additional information that may support patients in following their dietary plan and meeting their goals. This promoted the retention of the participants.

In each follow-up session, recurrent anthropometric measurements were taken. Likewise, data on physical activity and medication intake were assessed. Qualitative adherence data such as satisfaction with the diet, obstacles, and perceived changes, were also accessed.

At each visit, the data obtained from anthropometry and the biochemical studies corresponding to the previous consultation were given and explained to the subjects.

### 2.13. Biochemical Analysis

Blood samples were collected in a Serum tube, clot activator, silicone-coated interior and K2-EDTA Vacutainer^®^ tubes (Becton, Dickinson and Company, Franklin Lakes, NJ, USA). Blood samples were separated by centrifugation according to standard guidelines and were processed for measurement of glucose, total cholesterol (T-Chol), direct high-density lipoprotein cholesterol (HDL-C) and triglycerides (TG) at 3000 RPM, 15 min. A dry chemistry autoanalyzer (Vitros-350, Ortho Clinical Diagnostics, Raritan, NJ, USA) was used to process the samples. The principle of dry chemistry is based on reflectance spectrophotometry in which the reflectance of the materials is measured to give a reference standard for the comparison of the color of different samples. On the other hand, the low-density lipoprotein cholesterol (LDL-C) was calculated using the Friedewald formula as follows: [LDL-C = HDL-C (TG/5) (mg/dL)]. This formula is used if TG levels are less than 400 mg/dL, while very-low-density lipoprotein cholesterol (VLDL-C) was estimated by dividing total triglycerides by 5.

For insulin determination, the LIAISON Analyzer (LIAISON^®^, DiaSorin S.p.A; Saluggia, Italy) and the LIAISON^®^ Insulin reagent were used. This assay is a sandwich-type immunoassay based on the principle of chemiluminescence. In addition, for the analysis of glycosylated hemoglobin (HbA1c), samples were processed by an external certified laboratory (BIOSOMA, Clinic Laboratory; Guadalajara, Jalisco, Mexico). A total of 500 μL of whole blood was collected from an EDTA tube. Samples were stored at 4 °C and subsequently delivered to the corresponding laboratory. The quantification of HbA1c was performed using a chemiluminescent method.

The Homeostatic Model Assessment for Insulin Resistance (HOMA-IR) was calculated in Microsoft Excel, using the fasting serum glucose and insulin values of the participants in this study, according to the following formula: HOMA-IR = (Fasting insulin (µU/mL) × Fasting glucose (mg/dL))/405. A HOMA-IR index > 2.8 was used as the cut-off value to define insulin resistance [33].

The Triglyceride-Glucose (TyG) was calculated in Microsoft Excel, based on fasting serum glucose and triglyceride concentrations of the participants, according to the following formula: TyG index = Ln ((Triglycerides (mg/dL) × Fasting glucose (mg/dL))/2). A TyG index ≥ 4.68 was considered the diagnostic threshold for insulin resistance [34].

### 2.14. PBMC Isolation

In addition, another tube with EDTA for blood collection was prepared with Lymphoprep™ (Axis-shield, Dundee, Scotland) to isolate PBMC. In the first place, 4 mL of blood diluted 1:1 with 0.9% NaCl was carefully layered onto 4 mL of Lymphoprep™ and centrifuged at 800× *g*, 30 min, at room temperature. The band containing the PBMC was collected separately. The PBMC were washed with 10 mL of 0.9% NaCl, followed by a centrifugation cycle (250× *g*, 10 min, at room temperature) and supernatant removal. The washing step was repeated twice. The cells isolated were quantified by electrical impedance using HumaCount 30^TS^ (Human, Wiesbaden, Germany) normalized at 10 × 10^6^ cells/mL and resuspended in TRIzol^®^ reagent (Thermo Fisher Scientific Inc., Waltham, MA, USA), stored at −80 °C for a further real-time quantitative polymerase chain reaction (RT-qPCR) analysis, for immunoblotting/immunoprecipitation in RIPA buffer (Thermo Fisher Scientific Inc., Waltham, MA, USA).

### 2.15. Protein Isolation

After cell isolation, total protein from PBMC was immediately isolated following the next protocol: a standard buffer containing NaCl 150 mM, ethylenediaminetetraacetic acid (EDTA) 1 mM, Tris 25 mM, pH 8.0, glycerol 5% along with a protease/phosphatase inhibitor cocktail (Cat. A32961, Thermo Fisher Scientific Inc., Waltham, MA, USA). Total protein was obtained by centrifugation at 13,000× *g* for 30 min at 4 °C.

### 2.16. Serum Inflammatory Markers

Serum TNF-α, IL-6, interleukin 10 (IL-10), and interleukin 18 (IL-18) were measured at multiple time points (M0, M1 and M2) by using ProQuantum Immunoassay Kits TaqMan Applied Biosystems^TM^ (Thermo Fisher Scientific Inc., Waltham, MA, USA). The ProQuantum assay uses 2 oligonucleotide labels, which bind a pair of antibodies targeted against IL-6, IL-10, IL-18 and TNF-α, respectively. The PCR amplification uses the proximity ligation assay principle, and this interaction was measured by the amount of fluorescent signal, which is proportional to the amount of DNA produced after each amplification cycle. Each cytokine level was quantified by using a recombinant cytokine standard curve using the fluorescence threshold data at multiple time points.

### 2.17. Immunoprecipitation Analysis

In order to test the activation of the FFAR4 receptor, an immunoprecipitation assay from total isolated protein derived from PBMC was performed. For this purpose, Protein G SureBeads™ Magnetic Beads (Bio-Rad Laboratories, Inc., Hercules, CA, USA) were the procedure of choice, according to the manufacturer’s directions. This is intended to isolate the theoretical FFAR4/β-arrestin-2 complex using an anti-β-arrestin-2 antibody 1:1000 (Santa Cruz Biotechnology, Inc., Santa Cruz, CA, USA).

### 2.18. Western Blotting

The adjusted amounts of protein extracts were resuspended in SDS-containing Laemmli buffer, heated for 5 min at 95° C, and separated in a sodium dodecyl sulfate–polyacrylamide gel electrophoresis (SDS-PAGE) (6 and 10% percentage of gel concentration with molecular weight markers) under reducing conditions (2-mercaptoethanol). Then, the proteins were transferred to polyvinylidene difluoride (PVDF) membranes (Santa Cruz Biotechnology, Inc., Santa Cruz, CA, USA), followed by blocking at room temperature with non-fat dry milk and incubation with specific antibody against GPR120 1:1000 (Thermo Fisher Scientific Inc., Waltham, MA, USA) overnight at 4 °C. Afterward, membranes were incubated with either a secondary anti-rabbit (Thermo Fisher Scientific Inc., Waltham, MA, USA) or anti-mouse (LI-COR Biosciences NE, Lincoln, NE, USA) antibody 1:100,000 for 90 min at room temperature. Bands were detected using Immobilon^®^ Western Chemiluminescent HRP substrate (Merck Millipore, Burlington, MA, USA). The chemiluminescence was digitized using the C-Digit Blot Scanner (LI-COR Biosciences NE, Lincoln, NE, USA) and analyzed using Image Studio Digits Ver 5.2 processing software. Bands and density quantifications were normalized against β-actin (Santa Cruz Biotechnology, Inc., Santa Cruz, CA, USA) as a loading control.

To validate controls for the FFAR4/β-arrestin-2 immunoprecipitation and Western blot procedures, a pilot study was performed including both acute and chronic ω-3 PUFA supplementation as positive controls in healthy subjects [35].

In addition, the baseline samples from obese participants prior to ω-3 PUFA intervention exhibited minimal or undetectable FFAR4/β-arrestin-2 complex formation, serving as a functional negative reference for comparison. Together, these results confirm the specificity and reproducibility of the FFAR4/β-arrestin-2 immunoprecipitation assay under the experimental conditions applied in this study.

### 2.19. RNA Isolation and Gene Expression

The RNA isolation was performed with 4.5 × 10^6^ cells. First, in a 1.5 mL microtube, the sample was mixed with 1 mL of TRIzol^®^ reagent (Thermo Fisher Scientific Inc., Waltham, MA, USA). The tubes were kept on ice while the process was carried out. The mixture was incubated on ice for 3 min and then centrifuged at 11,600× *g* at 4 °C for 15 min. Afterwards, 200 μL of chloroform was added and vortexed vigorously for at least 15 s. The mixture was incubated on ice for 3 min and then centrifuged at 11,600× *g* at 4 °C for 15 min. Next, the aqueous supernatant was transferred to another microtube with 500 μL of cold absolute isopropanol. This was mixed with the pipette and incubated on ice for 20 min. After incubation, the mixture was centrifuged at 11,600× *g* at 4 °C for 10 min. To wash the RNA, the supernatant was discarded, and 1 mL of 75% ethanol was added and mixed with a pipette for a few seconds. The RNA was centrifuged at 10,000× *g* at 4 °C for 5 min. And finally, the supernatant was discarded to air-dry the RNA pellet for 15 min. The RNA pellet was dissolved in 20 μL Diethyl pyrocarbonate (DEPC)-treated water, quantified in a Multiskan SkyHigh Microplate Spectrophotometer (Thermo Fisher Scientific, Waltham, MA, USA). analyzed for quality on a 2% agarose gel and absorbance ratios of 260/280 nm and 260/230 nm.

**Figure 2:**
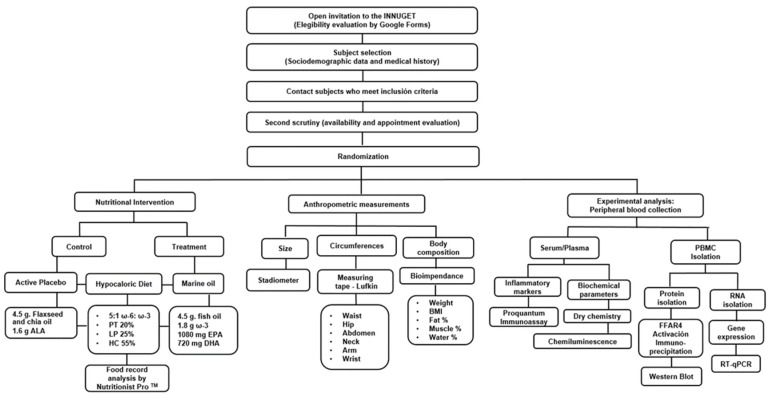
Methodological diagram of analyses and parameters.

A total of 1 μg of RNA was used to synthesize complementary DNA (cDNA) using Moloney murine leukemia virus reverse transcriptase (M-MMLV RT, Thermo Fisher Scientific Inc., Waltham, MA, USA) (conditions: annealing at 25 °C for 10 min; extension at 37 °C for 60 min; and final step at 95 °C for 10 min for inactivation). TaqMan Applied Biosystems^TM^ (Thermo Fisher Scientific Inc., Waltham, MA, USA) was used through specific probes: JUN N-Terminal Kinase (*JNK*)/Mitogen-Activated Protein Kinase 8 (*MAPK8*, ID:Hs01548508_m1, lot2175607) and the Inhibitor of Nuclear Factor Kappa B Kinase Subunit Beta (*IKKB*, ID:Hs01559460_m1, lot2151201) gene evaluation along with β-actin (*ACTB*, ID: Hs01060665_g1, lotP190412-017 G06) as a constitutively expressed gene in these cells according to standardization. The LightCycler^®^ 96 RealTime Polymerase Chain Reaction System (Hoffmann-La Roche, Basel, Switzerland) performed the analyses (conditions: pre-incubation at 50 °C for 120 s; initial denaturation at 95 °C for 600 s; followed by 40 cycles at 95° C for 15 s and at 60°C for 60 s). Relative mRNA expression was estimated by the 2−∆∆Cq method. Data were expressed as arbitrary units using the following calculation: [expression = 1000 × (2 − ΔCq) arbitrary units (AU)]. The complete methodological flux is shown in Figure 2.

### 2.20. Delivery of Results to Subjects

On every visit, every subject received a short report of their anthropometric and biochemical results in printed paper, and once the experimental phase concluded, patients were provided with their genetic results by email. Additionally, we scheduled group meetings to review the findings and clarify any questions that may arise.

### 2.21. Statistical Analysis

The primary endpoint was the change in FFAR4 activation in PBMC from baseline to final intervention and its correlation with ω-3 PUFA intake. Secondary endpoints (at week 4) included changes in inflammatory markers (serum and expression markers) and their correlations with FFAR4 activation. Exploratory endpoints encompassed anthropometric, biochemical and nutritional measures.

All statistical analyses were performed using the Statistical Package for Social Sciences (SPSS, version 22.0; IBM Corp., Armonk, NY, USA). Quantitative variables are presented as mean ± standard deviation (SD) or median (interquartile range), and qualitative variables as frequencies and percentages. Data normality was verified using the Shapiro–Wilk test.

Between-group comparisons were assessed with the independent Student’s *t*-test or Mann–Whitney U test, as appropriate. Within-group changes (baseline vs. post-intervention) were evaluated using the paired *t*-test or the Wilcoxon signed-rank test. For analyses involving more than two time points, repeated-measures ANOVA was applied, followed by Bonferroni post hoc tests for pairwise comparisons. When data did not meet parametric assumptions, the Friedman test was used as a non-parametric alternative. Correlations between continuous variables were analyzed using Pearson or Spearman coefficients. Significant correlations were further examined using linear regression analysis to explore the direction and strength of the associations. Categorical variables were compared with the χ^2^ test. Statistical significance was defined as *p* < 0.05.

## 3. Results

### 3.1. Recruitment and Participant Allocation

The recruitment for the nutritional intervention of the first (September–November 2021) and second phases (September–November 2022) of the study on the pro-resolution of chronic inflammation in obesity (EPICO) initially involved 236 applications. Of these, 152 were excluded due to failure to meet the inclusion criteria. Consequently, 84 patients were randomly assigned to one of two groups: active placebo or marine-derived ω-3, with 42 participants in each group.

During the nutritional follow-up, 21 participants were lost due to absenteeism (10 from the active placebo group and 11 from the marine ω-3 group) resulting in a total of 63 participants who completed the study. The main reasons for dropout were lack of attendance to follow-up visits and personal scheduling conflicts and non-intervention-related reasons. However, for data analysis, it was necessary to exclude 8 participants (3 from the active placebo group and 5 from the marine ω-3 group) due to the presence of metabolic disorders, non-permissible physiological conditions, or obesity-related comorbidities.

**Figure 3:**
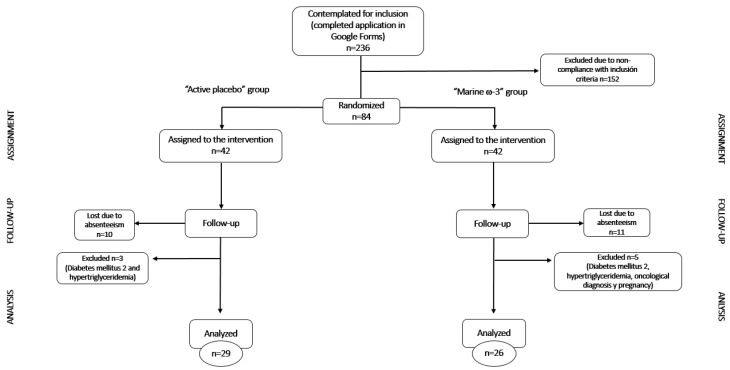
Flowchart of the Nutritional Intervention in the EPICO Project.

The final sample size included 55 participants: 29 in the active placebo group and 26 in the marine ω-3 group (Figure 3).

### 3.2. Demographic Data of the Study Groups

Table S5 presents the baseline demographic data of both study groups at the start of the intervention. The mean age in the active placebo group was 38.1 ± 10.1 years, while in the marine ω-3 group it was 38.2 ± 9.8 years. Regarding gender distribution, both groups showed similar proportions. In the active placebo group, 44.8% of participants were women, compared to 42.3% in the marine ω-3 group.

### 3.3. Anthropometric Data of the Study Groups

The comparison of baseline and final anthropometric variables, as well as differences between groups at the beginning and end of the intervention, is shown in Table 1. According to the within-group baseline vs. final analysis, significant changes were observed in the active placebo group for weight, BMI, body fat percentage (BFP), and waist and abdominal circumferences (*p* < 0.01). Similarly, in the marine ω-3 group, significant differences were found in weight, skeletal muscle mass (SMM), BMI, and waist circumference (*p* < 0.01).

Between-group analyses revealed no significant differences in anthropometric variables at baseline, indicating that both populations had comparable characteristics at the start of the study. Additionally, the table presents the comparison of changes (delta) in body composition parameters from the beginning to the end of the intervention. The data showed no significant differences in any of the variables between groups.

### 3.4. Biochemical Results of the Study Groups

Table 2 presents the comparison of baseline and final biochemical parameters, as well as differences between groups at the beginning and end of the intervention. Within-group baseline vs. final analyses revealed significant changes in the active placebo group for insulin (*p* = 0.02), HbA1c (*p* = 0.02), TyG index (*p* = 0.04), HDL-C (*p* < 0.01), LDL-C (*p* < 0.01), and VLDL-C (*p* = 0.04). In the marine ω-3 group, significant differences were observed in insulin levels (*p* = 0.01), HbA1c (*p* = 0.01), TyG index (*p* < 0.01), triglycerides (*p* = 0.04), HDL-C (*p* < 0.01), and VLDL-C (*p* = 0.01). Regarding between-group comparisons, no significant differences were found in biochemical variables at baseline. In addition, the table summarizes the comparison of changes (deltas) in biochemical variables at the end of the intervention. No significant differences were observed in these parameters.

From this point onward, the results from the second cohort of the EPICO project (previously described in the methodology section) are presented. This cohort included a total of 14 participants, who were randomized into the active placebo and marine ω-3 groups, with n = 7 participants per group.

### 3.5. Dietary Results (Complementary Cohort)

Statistical analyses of dietary variables were conducted specifically for the complementary cohort (n = 14 from the second phase of EPICO recruitment, n = 7 per group). The subsequent results present a comparison of macronutrient intake and consumption of ω-3 from both dietary sources and supplementation in the study groups. Table 3 shows the analyses of dietary variables between baseline and final time points for each study group. No significant differences were observed in macronutrient intake.

However, ω-3 PUFA consumption was significantly higher in the marine ω-3 group (*p* = 0.01), particularly for EPA (*p* < 0.01) and DHA (*p* = 0.01), compared to the active placebo group at the end of the intervention. Additionally, both the active placebo group (*p* = 0.04) and the marine ω-3 group (*p* < 0.01) showed an increase in ω-3 intake and a reduced ω-6:ω-3 ratio in the diet after the intervention (*p* < 0.01 and *p* = 0.04, respectively). Finally, only the marine ω-3 group demonstrated a significant increase in EPA (*p* < 0.01) and DHA (*p* < 0.01) consumption compared to baseline intake (Table 4).

Regarding overall adherence to the intervention, which included total capsule consumption, dietary intake, and the ω-6:ω-3 PUFA ratio, similar patterns were observed between the groups. In the active placebo group, adherence in the first month reached 68.0 ± 19.3%, while in the marine ω-3 group, it was 79.1 ± 27.7%. By the final time (second month), adherence declined in both groups, with a mean of 66.6 ± 8.3% in the active placebo group and 69.4 ± 27.7% in the marine ω-3 group. No significant differences in adherence levels were found within groups (intragroup) or between groups (intergroup) during either month of the intervention (Table 5). Overall, these results indicate satisfactory adherence across the study groups.

### 3.6. FFAR4 Activation Analysis (Complementary Cohort)

#### 3.6.1. FFAR4 Baseline Activation (Complementary Cohort)

**Figure 4:**
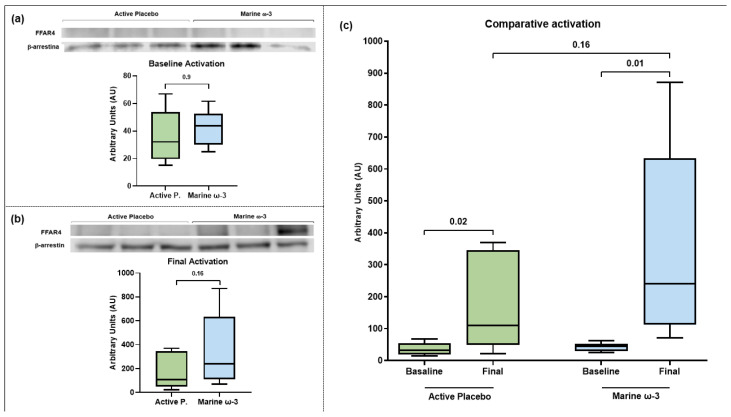
Evaluation of FFAR4 activation by immunoprecipitation of βarr-2 and Western blot. FFAR4 detection was performed on a PVDF membrane, and band density was normalized using βarr-2 as a loading control. The graphs represent the numerical values (median, IQR, and minimum-maximum) of the densitometric analysis of the bands shown above. Mann–Whitney U test. (**a**) Baseline comparison between groups, (**b**) Final comparison between groups and (**c**) Comparative activation overview.

Immunoprecipitation and Western Blot assays were performed to detect the FFAR4 receptor. Figure 4a presents the immunoblot analysis at the beginning of the intervention. Overall, densitometric analysis revealed a median expression in both groups exceeding 40 arbitrary units. In the active placebo group, the interquartile range (IQR) ranged from 25 to 65 units, whereas in the marine ω-3 group, it ranged from 35 to 60 units. Therefore, baseline FFAR4 expression levels did not show statistically significant differences between groups.

#### 3.6.2. FFAR4 Activation at the End of Intervention (Complementary Cohort)

Regarding receptor expression levels at the end of the intervention (two months), Figure 4b presents the results obtained from the immunoprecipitation and Western Blot assays. Densitometric analysis of the bands showed a median FFAR4 expression below 100 arbitrary units in the active placebo group, whereas in the marine ω-3 group, it exceeded 200 arbitrary units. Additionally, the IQR in the active placebo group varied between 50 and 350 units, while in the marine ω-3 group, it ranged from 100 to 600 units. However, no significant differences were observed in the final FFAR4 expression levels between the study groups.

Figure 4c presents a comparative FFAR4 activation overview between the baseline and final time points in both study groups. Although no significant differences were observed between groups at the end of the two-month intervention, an intra-group comparison between baseline and final time points was also performed. In this regard, both the active placebo group (*p* = 0.02) and the marine ω-3 group (*p* = 0.01) showed a significant increase in FFAR4 expression levels following the nutritional intervention.

### 3.7. Correlation Between PUFA Intake and FFAR4 Activation (Complementary Cohort)

The correlation analysis between FFAR4 activation and the consumption of major PUFA (ω-3, EPA, DHA, ALA, and ω-6) was performed. In this regard, EPA intake showed a positive correlation with FFAR4 activation (*p* = 0.04). Conversely, the results indicated that higher linoleic acid (LA) and ω-6 intake correlated negatively with FFAR4 activation (*p* = 0.01) in the marine ω-3 group (Table 6).

**Figure 5:**
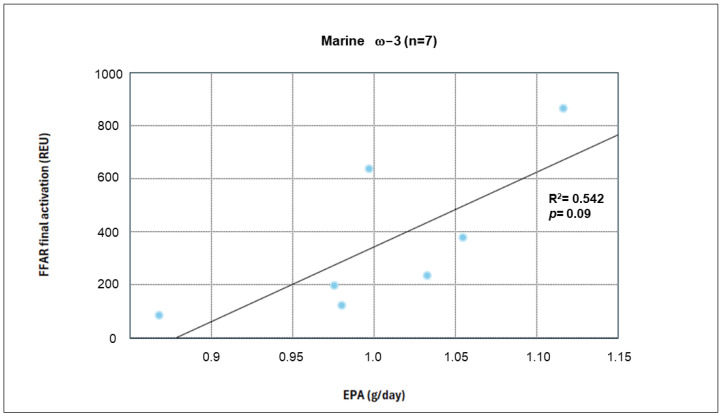
Linear Regression of EPA Intake and FFAR4 Activation.

Among the reported correlations, EPA showed a moderate but non-significant correlation in the linear regression analysis (R^2^ = 0.542, *p* = 0.09) (Figure 5).

### 3.8. Relative Expression of Target Genes (Complementary Cohort)

#### 3.8.1. Gene Expression of JNK

**Figure 6:**
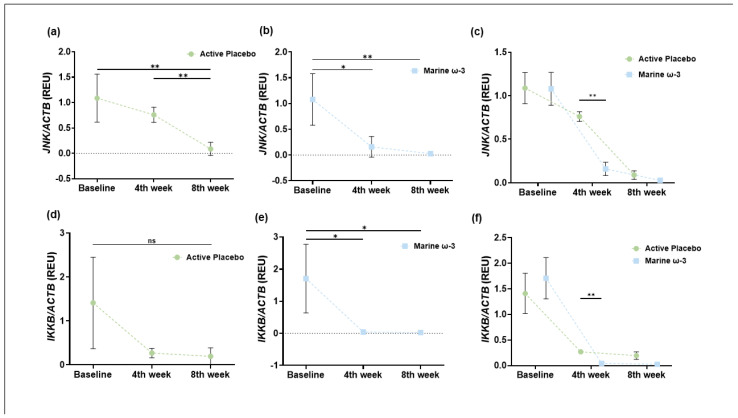
Relative expression units of *JNK* and *IKKB* in the active placebo group (**a**,**d**) and marine ω-3 (**b**,**e**) or comparative expression overview (**c**,**f**) during the intervention. Data are expressed as mean and standard deviation (SD). Repeated-measures ANOVA, Bonferroni post hoc analysis. * *p* < 0.05 and ** *p* < 0.01.

The expression of key genes in the FFAR4 pathway was analyzed. First, the expression of the *JNK* gene was evaluated at different time points within each group. The active placebo group exhibited a progressive decrease (Figure 6a), while the marine ω-3 group also showed a reduction in *JNK* expression, particularly at month 1 of the intervention (Figure 6b). Additionally, Figure 6c presents a comparison between the intervention groups, revealing a statistically significant change in *JNK* mRNA expression at month 1 of the intervention.

#### 3.8.2. Gene Expression of IKKB (Complementary Cohort)

Subsequently, *IKKB* expression levels were compared. The active placebo group showed no differences in the levels of this inflammatory marker (Figure 6d). However, the marine ω-3 group exhibited a significant reduction at months 1 and 2 (final) compared to baseline (Figure 6e). Furthermore, Figure 6f presents the between-group comparison of *IKKB* gene expression, revealing a statistically significant decrease in the marine ω-3 group at month 1 compared to the active placebo group.

### 3.9. Sub-Analysis of Global FFAR4 Activation in the Study Subjects (Complementary Cohort)

**Figure 7:**
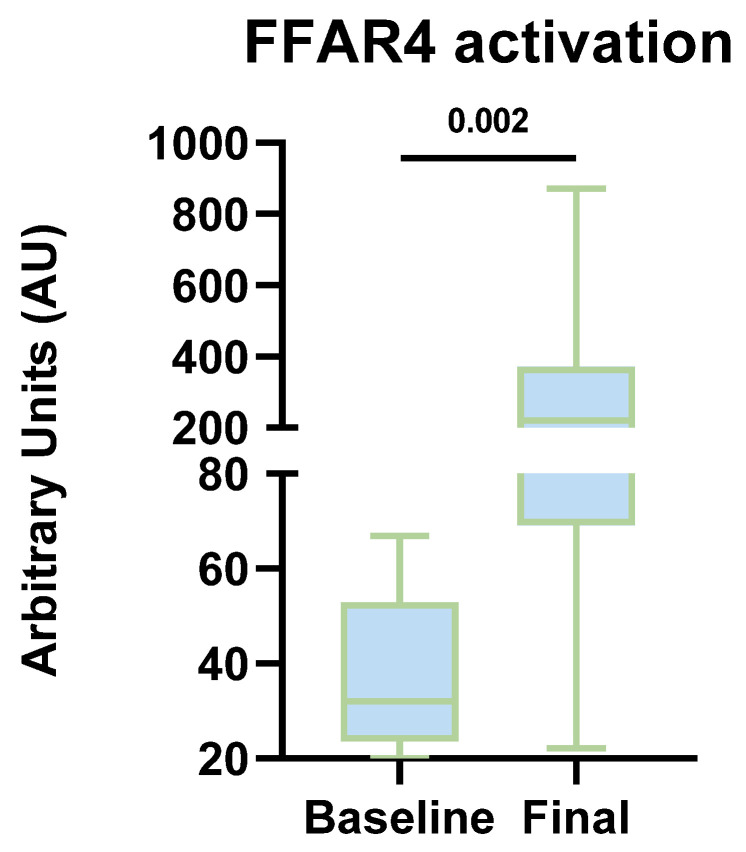
Comparative FFAR4 activation at baseline and final time points in the complementary cohort (n = 14). The graphs represent the numerical values (median, IQR, and minimum-maximum) obtained from the densitometric analysis of the Western Blot. Mann–Whitney U test.

A series of sub-analyses were conducted to assess global FFAR4 receptor activation in all the study subjects of the complementary cohort (n = 14) at both the beginning and end of the intervention. These analyses were primarily driven by the observed increase in ω-3 PUFA intake and the achievement of an optimal ω-6:ω-3 ratio. In this regard, Figure 7 illustrates a marked increase in FFAR4 activation at the final time.

#### Correlation Between Global FFAR4 Activation and Inflammatory Markers Expression (Complementary Cohort)

Table 7 presents the correlation analysis results between global FFAR4 activation in the complementary cohort (n = 14) and the expression of the inflammatory markers *JNK* and *IKKB*. A statistically significant negative correlation was observed for *JNK* expression at the end of the intervention.

**Figure 8:**
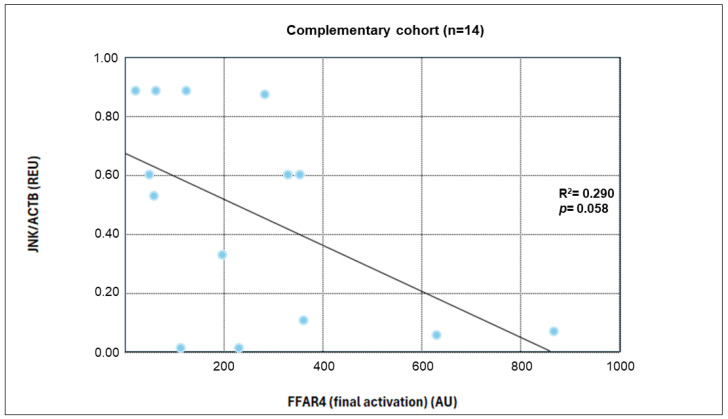
Correlation Between FFAR4 Receptor Activation Levels and *JNK* Inflammatory Marker Expression.

Subsequently, a linear regression analysis was performed between receptor activation levels and *JNK* expression, yielding an R^2^ value of 0.29. However, this finding showed a *p*-value of 0.058 (Figure 8).

### 3.10. Inflammatory Parameter Results (Cytokines)

Table 8 presents the results of the serum inflammatory marker evaluation. The active placebo group showed a statistically significant reduction in TNF-α and IL-18 levels (*p* < 0.05) at the end of the intervention. Meanwhile, the marine ω-3 group exhibited a significant decrease in TNF-α, IL-6, and IL-18 levels, and a significant increase in IL-10 (*p* < 0.05).

### 3.11. Correlation Analysis of FFAR4 Activation and Serum Inflammatory Markers (Complementary Cohort)

Correlation analyses were performed to evaluate the linear relationship between the serum inflammatory markers and FFAR4 receptor activation at the end of the intervention. Table 9 shows a negative correlation coefficient for most parameters in both groups. However, a statistically significant negative correlation was observed for TNF-α levels and a positive correlation for IL-10 levels only in the marine ω-3 group.

**Figure 9:**
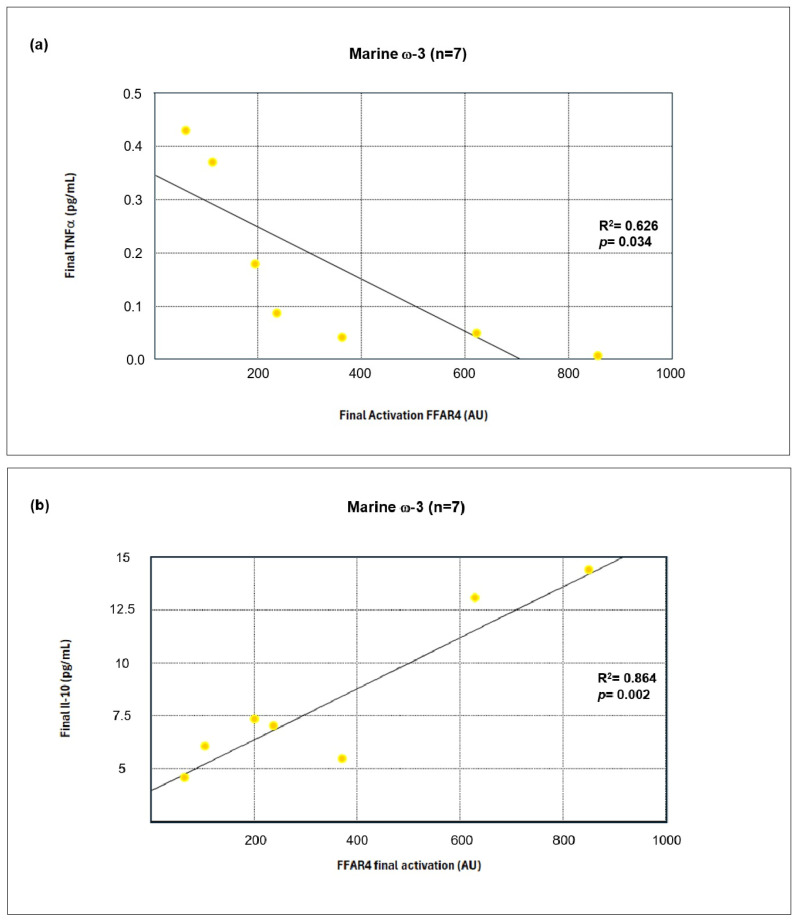
Linear Regression of FFAR4 activation and TNF-α (**a**) or IL-10 (**b**) levels in the Marine ω-3 group at the End of the Intervention.

Subsequently, a linear regression model was performed on the variables that showed statistically significant correlations. Figure 9a,b display the statistical confirmation of the strong relationship between these variables in the marine ω-3 group. Both TNF-α and IL-10 levels again showed statistical significance (*p* < 0.05).

## 4. Discussion

Given the multifactorial etiology of obesity, understanding its cellular and molecular mechanisms is crucial. Recent attention has focused on the interplay between physiological components, inflammatory markers, and expression of intermediary molecules in response to ω-3 PUFA intake [36]. This study bridges nutrition and molecular biology within translational nutrigenomics, demonstrating changes in anthropometric, dietary, biochemical, and inflammatory parameters in 55 individuals with obesity following ω-3 supplementation.

The following sections will sequentially discuss all relevant aspects:

In relation to the effect of ω-3 on anthropometric parameters, the ω-3-supplemented intervention led to improvements in body composition, with reductions in weight, BMI, body fat percentage, and waist and abdominal circumferences observed in both groups. However, no significant differences emerged between the marine ω-3 and active placebo groups, consistent with previous studies using 1–4 g/day of EPA and DHA alongside caloric restriction [37,38,39]. These outcomes are primarily attributed to the caloric restriction and balanced macronutrient intake, including a controlled ω-6:ω-3 ratio, implemented in both groups. Metabolic adaptations such as enhanced glycogenolysis and β-oxidation likely contributed to these effects [40,41]. Notably, a 70–80% dietary adherence rate was sufficient to achieve these changes [42].

With respect to biochemical parameters, the ω-3 PUFA-supplemented intervention resulted in a significant reduction in triglyceride and VLDL-C levels in the marine ω-3 group, consistent with previous studies [43]. Although the reduction in triglyceride levels in the active placebo group was not statistically significant, the observed trend likely reflects favorable metabolic responses driven by dietary modifications and increased intake of EPA and DHA through the diet, in combination with nutritional counseling. Mechanistically, this effect is associated with the inhibition of triglyceride synthesis through downregulation of SREBP-1c (sterol regulatory element-binding protein 1c) and DGAT (diacylglycerol acyltransferase), both key enzymes in triglyceride synthesis and transport. Meta-analyses support that ω-3 PUFAs primarily reduce triglycerides without significantly affecting other lipid parameters [44]. In line with this, the American Heart Association (AHA) recommends marine ω-3 supplementation, as 1 g/day of EPA and DHA can reduce triglyceride levels by 5–10% [39,40,41,42,43].

The increase in LDL-C observed in both groups may indicate a shift toward larger, less atherogenic LDL-C particles, as previously described in studies using high-dose DHA, which reported an 8% rise in LDL-C associated with reduced cardiovascular risk. This effect may be attributed to ω-3 PUFA-mediated regulation of genes involved in triglyceride metabolism—SREBP-1c, FAS (fatty acid synthase), DGAT, PPAR-α (peroxisome proliferator-activated receptor alpha), and ACO (acyl-CoA oxidase)—leading to lower VLDL-C synthesis and circulating triglycerides. As a result, CETP (cholesteryl ester transfer protein)-mediated transfer of triglycerides from VLDL-C to LDL-C is reduced, maintaining larger LDL-C particle size. Moreover, the decrease in smaller VLDL-C particles may increase competition with LDL-C for hepatic LDL receptor uptake, slightly elevating circulating LDL-C levels. Nevertheless, the shift toward larger LDL-C particles suggest a beneficial remodeling of the lipid profile induced by ω-3 PUFA intake [45].

Additionally, HDL-C levels declined in both groups, possibly due to caloric restriction and the absence of significant changes in physical activity, as most participants reported low or low-intensity exercise on the IPAQ. Evidence indicates that low physical activity limits HDL-C concentration and functionality by reducing lipoprotein lipase activity, pre-β-HDL formation, and reverse cholesterol transport [46]. Furthermore, the decrease in HDL-C may reflect increased cellular uptake during macrophage polarization toward an anti-inflammatory phenotype, which supports homeostasis through enhanced IL-10 secretion [43,44,45].

Regarding glucose homeostasis, a significant reduction in fasting insulin levels was observed in both groups, while the marine ω-3 group also showed a slight, non-significant decrease in HOMA-IR. These findings align with prior studies using comparable DHA and EPA doses, though between-group differences remained non-significant. The improvements may be mediated by EPA’s anti-inflammatory effects and DHA-induced activation of PPARγ (peroxisome proliferator-activated receptor gamma), which enhances adiponectin secretion and insulin sensitivity [47]. A slight increase in fasting glucose was also detected, possibly due to caloric restriction-induced gluconeogenesis and substrate redistribution, as previously reported in individuals with diabetes mellitus 2 supplemented with ω-3 [48]. However, participants in this study did not present relevant alterations in glucose metabolism, unlike populations with diabetes mellitus 2, which may explain the modest magnitude of change. Additionally, the literature reports mixed outcomes on glucose, with some trials showing no effect [49,50]. Interpretation of these results is further limited by punctual glucose measurement and variable adherence. Regarding the TyG index, although no significant differences between groups were observed, both groups exhibited a reduction. Notably, this is the first clinical trial to evaluate TyG following dietary ω-3 supplementation—a marker increasingly associated with cardiometabolic risk and NASH [51,52,53,54]. Overall, glycemic responses to ω-3 appear modest in metabolically stable individuals and are likely influenced by dose, intervention duration, adherence, and especially the EPA:DHA ratio, which is critical depending on the intended metabolic outcome [55].

Concerning the effect of the nutritional intervention on FFAR4 activation, the ω-3 PUFA, specifically EPA and DHA, have demonstrated anti-inflammatory effects partially mediated by the FFAR4 receptor. Upon activation, this receptor promotes various anti-inflammatory mechanisms, and its role has been explored in previous studies. In this project, we evaluated FFAR4 activation in PBMC, based on evidence of its expression in peripheral adipose tissue macrophages and Kupffer cells, where its activation is associated with polarization toward an anti-inflammatory M2 phenotype via TAK1 inhibition [56,57]. Consistently, Oh et al. demonstrated that DHA exposure increases FFAR4 expression in RAW 264.7 macrophages and 3T3-L1 adipocytes (100 µM DHA), findings that align with experiments in HEK293 cells [58]. Moreover, a triple-blind clinical trial conducted in Brazil by Batista et al. assessed both acute and chronic FFAR4 activation following ω-3 supplementation in PBMCs from patients with Non-Alcoholic Fatty Liver Disease (NAFLD) and overweight. Their findings showed that monocytes and lymphocytes are sensitive to inflammatory proteins and respond to fatty acid levels by regulating the expression of genes associated with immunity and lipid metabolism [59]. In this context, identifying FFAR4 activation in humans raises the possibility that the effects reported in in vitro and in vivo models may also be observed in humans supplemented with ω-3 PUFAs.

When evaluating FFAR4 activation in the complementary cohort, we identified the presence of this receptor at both the beginning and end of the intervention in both groups. Furthermore, increased expression and activation levels were observed in each group (measured in arbitrary units). The findings of this project provide mechanistic evidence of activation in a peripheral environment, which, being less controlled, offers a more relevant approximation of what might occur in adipose tissue in obese patients. Although between-group comparisons at the end of the intervention did not reveal statistically significant differences in FFAR4 activation, several factors could have influenced these findings. First, the small sample size contributed to high data variability, as evidenced by the wide dispersion measures (standard deviation). Additionally, dietary intake analysis revealed an increase in EPA and DHA consumption in the active placebo group as well, leading to enhanced activation in both groups and making it difficult to detect significant differences at the end of the intervention, given the priority of minimizing health risks.

The analysis of FFAR4 activation has progressively evolved based on prior reports that served as a reference for our study. Da Young et al. conducted a pioneering FFAR4 study, performing co-immunoprecipitation analyses that identified the FFAR4–βarrestin-2 complex in RAW 264.7 cells stimulated with 100 μM DHA. They also performed co-transfection in HEK293 cells, where they detected internalization of the FFAR4–βarrestin-2 heterodimer 30 min after DHA stimulation [60]. Finally, Dátilo et al. confirmed these findings in tissues such as the retina and colon in a murine model subjected to an eight-week linseed oil supplementation (100 g/kg of food), which led to elevated FFAR4 levels [56,61]. In this regard, our results provide human-based evidence showing that increased EPA and DHA intake promotes FFAR4 activation in both groups.

A key factor contributing to the robustness of our results was the selection of the active placebo. It has been reported that inappropriate use of ω-6 and ω-9 sources as placebos can lead to either overestimation or underestimation of the beneficial effects of ω-3, respectively. Therefore, the use of an ALA-based placebo in the active placebo group facilitated the beneficial effects of the nutritional intervention and dietary EPA and DHA intake in both study groups.

Additionally, a crucial aspect of the activation phenomenon was the recorded change in the ω-6:ω-3 intake ratio, which reached an ideal proportion of 5:1 at the end of the intervention in both groups. This ratio has been described as essential for receptor activation, as a balanced proportion of these PUFA is more influential than total ω-3 intake [62]. This is due to the competitive inhibition exerted by high ω-6 consumption on the ω-3 metabolic pathway.

To date, no published evidence exists regarding FFAR4 expression and activation levels in humans. Our findings provide the first reported data on FFAR4 activation levels in PBMCs from patients following a diet supplemented with ω-3 PUFA. This suggests the potential for FFAR4 activation to extend from monocytes to resident adipose tissue macrophages, which could have implications in promoting pro-resolving mechanisms and improving insulin sensitivity, thereby modulating the inflammatory response in obesity.

The scope of our study allows us to recognize relevant dietary factors that should be considered in future research: extending the intervention duration and increasing sample size to obtain findings that further complement our results, addressing the questions that arise from the present study.

In connection with gene expression, analysis of target genes showed a decrease in the expression of *IKKB* and *JNK*, both of which are critical components of the MAPK (Mitogen Activated Protein Kinases) and NFκB inflammatory pathways, respectively. These kinases are key mediators of NFκB activation and nuclear translocation, thus playing critical roles in proinflammatory signaling. The downregulation observed in our study supports previous findings where FFAR4 activation reduced the expression and phosphorylation of JNK and IKKB. For example, in RAW 264.7 macrophages, FFAR4 stimulation—via synthetic agonist GM9508 and DHA—resulted in lower abundance and phosphorylation of these proteins. These effects were confirmed using FFAR4 knockdown and knockout models, indicating that receptor-mediated signaling suppresses inflammatory gene expression [60]. Our results suggest a similar mechanism may occur in vivo following ω-3 PUFA supplementation, with functional repression of JNK and IKKB potentially contributing to the anti-inflammatory effect observed.

When considering the analysis of serum inflammatory markers, low-grade chronic inflammation is a hallmark of obesity, driven by adipocyte–immune cell interactions and increased secretion of proinflammatory cytokines such as TNF-α, IL-6, and IL-18, which contribute to insulin resistance and adipose tissue dysfunction. In this study, the dietary intervention led to significant reductions in TNF-α and IL-18 in the active placebo group, and TNF-α, IL-6, and IL-18 in the marine ω-3 group. These changes suggest both caloric restriction and marine ω-3 PUFAs exert anti-inflammatory effects, with greater impact observed in the supplemented group.

TNF-α was significantly reduced in both groups, consistent with its known role in impairing insulin signaling and activating NFκB. The inverse correlation between *FFAR4* activation and TNF-α levels supports the hypothesis that FFAR4 suppresses proinflammatory pathways [63,64]. Similarly, IL-6, primarily secreted by adipose tissue, decreased only in the marine ω-3 group. This aligns with findings showing that DHA inhibits IL-6 expression through FFAR4, an effect abolished when the receptor is silenced [65].

The reduction in IL-18, a cytokine linked to both metabolic regulation and inflammation, was observed in both groups. IL-18 promotes NFκB and MAPK activation and is associated with insulin resistance, atherogenesis, and metabolic syndrome [66,67]. ω-3 PUFAs, particularly EPA and DHA, inhibit NLRP3 inflammasome activation—an essential mechanism for IL-18 maturation—through interactions with FFAR4 and β-arrestin-2 [68,69,70]. Thus, its reduction may reflect inflammasome suppression via FFAR4-mediated signaling.

In contrast, IL-10, an anti-inflammatory cytokine that regulates macrophage activity and promotes M2 polarization, increased significantly only in the marine ω-3 group. This may be mediated by FFAR4-dependent upregulation of adiponectin and regulatory T-cell activity, favoring an immunoregulatory microenvironment [71,72,73]. The positive correlation between IL-10 and FFAR4 activation further supports its anti-inflammatory role [74].

Together, these results demonstrate a systemic shift toward inflammation resolution, especially in the ω-3 group. Correlation analyses suggest that FFAR4 activation is inversely associated with proinflammatory markers and positively associated with immunomodulatory cytokines. Although the present findings reveal significant correlations between ω-3 PUFA intake, FFAR4 activation, and the expression of inflammatory markers, these results should be interpreted as associative. The regression and correlation analyses performed here emulate potential mechanistic relationships. Nevertheless, these insights provide a valuable framework for understanding how FFAR4 activation may be linked to downstream inflammatory signaling pathways. Future studies using controlled in vitro or ex vivo models are needed to validate the mechanistic interactions underlying the observed associations.

This is the first clinical study to demonstrate FFAR4 activation in human PBMCs following dietary supplementation with EPA and DHA, providing valuable translational insight into the systemic anti-inflammatory effects of marine ω-3 PUFA. Conducted within a Nutritional Genomics framework, the study integrated anthropometric, dietary, biochemical, gene expression, and protein-level data, enabling a comprehensive, multidimensional assessment of the intervention’s impact. The combination of molecular analyses with a controlled nutritional strategy underscores the clinical relevance of ω-3 PUFAs as functional bioactive compounds in precision approaches for managing chronic inflammation in obesity.

The study included some limitations that should be considered. Initially, the small sample size in which the receptor activation analysis was performed is a significant factor, and it would therefore be advisable to replicate the analysis in a larger sample. Another important aspect is the level of adherence to the semi-personalized diet, which generates variability in energy intake and sources of fatty acids. Additionally, there was variability in physical activity levels both within and between groups. However, these two variables were assessed using validated tools to ensure appropriate comparisons. Another limitation of the study was the small difference in ω-3 PUFA content between the marine ω-3 group (600 mg) and the active placebo group (550 mg), due to the available doses provided by the manufacturers. Although this difference is minimal and likely does not significantly affect the overall results, it was considered when interpreting the study findings. Nevertheless, both doses fall within the effective range for ω-3 supplementation, which ensured a solid comparison for the trial. It is important to note that several correlation and regression analyses presented in this study were exploratory in nature and based on a reduced sample size (n = 7 per group). While these analyses provided valuable insights particularly regarding associations between PUFA intake, FFAR4 activation, and inflammatory markers, the statistical power was limited. Therefore, these results should be interpreted with caution and considered hypothesis-generating rather than confirmatory. Future studies with larger and more homogeneous populations are required to validate these findings and further elucidate the underlying molecular mechanisms. Finally, an additional aspect to consider in the interpretation of this trial is the use of alpha linolenic acid (ALA) as an active placebo. ALA can undergo endogenous desaturation and elongation to form ω-3 PUFA such as EPA and DHA. However, this metabolic conversion in humans is known to be very limited. typically below 10–15% for EPA and 0.5–1% for DHA, and influenced by factors such as sex, hormonal status, and genetic variability in Fatty Acid Desaturase 1 and 2 (*FADS1* and *FADS2*) genes [48,75]. Given these low conversion rates and the duration of the intervention, it is unlikely that ALA substantially increased circulating EPA or DHA levels or exerted a meaningful effect on FFAR4 activation or inflammatory markers. Therefore, ALA served as a physiologically appropriate active placebo, allowing comparable dietary fatty acid profiles between groups while minimizing potential confounding associated with ω-6–rich vegetable oils. Nevertheless, the partial metabolic conversion of ALA to ω-3 PUFA cannot be entirely excluded and was considered a limitation of the study.

## 5. Conclusions

This clinical trial provides the first translational evidence of FFAR4 receptor activation in PBMC following dietary supplementation with marine ω-3 PUFA. Importantly, this is also the first study to compare the effects of marine EPA and DHA PUFA versus plant-derived ALA on FFAR4 activation and inflammatory responses in humans. FFAR4 activation was associated with favorable modulation of pro- and anti-inflammatory markers, as well as improvements in metabolic parameters. These findings reinforce the potential of marine ω-3 supplementation as an adjunctive strategy for mitigating chronic inflammation in obesity and position FFAR4 as a relevant target for future clinical nutrition and immunometabolic research.

## Figures and Tables

**Table 1:** Anthropometric data of the study groups: intragroup and intergroup comparison.

	Active Placebo Group (n = 29)				Marine ω-3 Group (n = 26)				
Anthropometrics									
Variable	Baseline	Final	Δ	*p* ^1^	Baseline	Final	Δ	*p* ^2^	*p* ^3^
Weight (Kg)	98.3 ± 16.2 *	95.8 ± 15.3	−2.5 ± 3.2 *	**<0.01**	94.7 ± 15.3	91.3 ± 14.3	−3.3 ± 3.3 *	**<0.01**	0.38
SMM (Kg)	31.2 ± 6.0	31.0 ± 5.9	−0.3 ± 1.1	0.28	30.6 ± 6.8 *	30.2 ± 6.8 *	−0.4 ± 1.5	**<0.01**	0.35
BMI (Kg/m^2^)	34.7 ± 3.6	34.2 ± 3.8	−0.8 ± 1.0	**<0.01**	33.6 ± 3.1 *	32.5 ± 2.7 *	−1.1 ± 1.1 *	**<0.01**	0.36
BFP (%)	44.4 ± 5.8	43.1 ± 6.9	−1.2 ± 2.0 *	**<0.01**	41.1 ± 9.1 *	40.5 ± 5.6	−0.6 ± 7.7 *	0.06	0.80
Waist C. (cm)	105.9 ± 13.0	101.3 ± 12.9	−3.8 ± 2.3	**<0.01**	103.0 ± 11.5	99.1 ± 10.4	−4.7 ± 3.2 *	**<0.01**	0.52
Hip C. (cm)	118.0 ± 11.7	116.6 ± 10.1	−0.9 ± 5.6 *	0.42	115.4 ± 8.1	113.5 ± 7.7	−1.3 ± 3.9	0.14	0.95
Abdominal C. (cm)	116.3 ± 14.0	110.4 ± 14.2	−5.6 ± 8.6 *	**<0.01**	112.6 ± 12.2	107.0 ± 10.6	−6.0 ± 10.9 *	**0.02**	0.85

The variables are expressed as mean and standard deviation. BMI: body mass index, C: Circumference, BFP: body fat percentage. *t* test for two independent samples/Mann–Whitney U. The baseline comparison of the variables between the groups was carried out and no statistically significant differences were found at the beginning of the intervention. The *t* test for two independent samples/Mann–Whitney U was performed according to the normality of the variables. * Non-parametric variables. *p*^1^ Comparison between final vs. baseline in the active placebo group (intragroup). *p*^2^ Comparison between final vs. baseline of the ω-3 marine group (intragroup). *p*^3^ Comparison between the groups (intergroup: Active placebo group vs. marine ω-3 group). Bold is used to point out statistical significance. Same applies for the rest of the tables.

**Table 2:** Biochemical variables in the study groups: intragroup and intergroup comparison.

	Active Placebo Group (n = 29)				Marine ω-3 Group (n = 26)				
Biochemical Variables									
Variable	Baseline	Final	Δ	*p* ^1^	Basal	Final	Δ	*p* ^2^	*p* ^3^
Glucose (mg/dL)	93.4 ± 7.9 *	94.1 ± 11.3 *	0.9 ± 10.1 *	0.37	96.9 ± 10.6 *	100.4 ± 10.2 *	3.2 ± 13.9 *	0.30	0.66
Insulin (mg/dL)	17.3 ± 8.7 *	13.5 ± 6.4 *	−2.8 ± 6.7	**0.02**	19.7 ± 11.1 *	13.2 ± 7.6 *	−5.2 ± 11.1	**0.01**	0.70
Hb1Ac (%)	5.3 ± 0.3 *	5.6 ± 0.2 *	0.04 ± 0.49	**0.02**	5.4 ± 0.5 *	5.7 ± 0.3 *	0.1 ± 0.47	**0.01**	0.84
HOMA-IR	4.0 ± 2.0 *	3.2 ± 1.6 *	−0.6 ± 1.6	0.06	4.6 ± 2.5 *	3.2 ± 1.8 *	−0.9 ± 2.6	0.07	0.77
HOMA-IR n (%)	19 (65.5)	12 (41.4)	−7 (−24.1)	0.37	17 (68.0)	13 (52.0)	−4 (−16)	0.37	-
TyG	4.7 ± 0.2	4.6 ± 0.2	4.7 ± 0.1	**0.04**	4.8 ± 0.2	4.7 ± 0.2	4.8 ± 0.1	**<0.01**	0.43
TyG n (%)	18 (62.1)	11 (37.9)	−7 (−24.2)	0.37	18 (72.0)	15 (60.0)	−3 (12.0)	0.50	-
Tryglicerides (mg/dL)	154.9 ± 74.2 *	136.2 ± 60.5 *	−16.5 ± 44.8 *	0.05	184.8 ± 73.1 *	149.4 ± 75.3 *	−28.1 ± 60.8 *	**0.01**	0.27
T-chol (mg/dL)	158.6 ± 31.4 *	160.4 ± 33.2 *	0.8 ± 13.4 *	0.96	170.6 ± 22.2 *	162.2 ± 34.6 *	−7.0 ± 24.4 *	0.17	0.38
HDL-C (mg/dL)	39.9 ± 8.3	36.0 ± 6.5	−3.7 ± 5.0 *	**<0.01**	38.5 ± 10.3	33.8 ± 7.7	−3.5 ± 5.7 *	**<0.01**	0.62
LDL-C (mg/dL)	87.7 ± 22.6	97.6 ± 23.4	8.2 ± 15.4 *	**<0.01**	94.2 ± 19.4	102.7 ± 16.6	8.0 ± 17.2 *	0.07	0.59
VLDL-C (mg/dL)	31.0 ± 14.8 *	27.2 ± 12.1 *	−3.3 ± 8.8 *	**<0.01**	37.9 ± 14.8 *	29.8 ± 15.1 *	−6.4 ± 12.6 *	**0.01**	0.20

The variables are expressed as mean ± standard deviation. HbA1c: Glycated hemoglobin; HOMA-IR: Homeostatic Model Assessment for Insulin Resistance; IR: Insulin Resistance; TyG: Triglyceride-Glucose Index; T-chol: Total Cholesterol; HDL-C: High-Density Lipoprotein Cholesterol; LDL-C: Low-Density Lipoprotein Cholesterol; VLDL-C: Very-Low-Density Lipoprotein Cholesterol. Paired *t*-tests or Wilcoxon tests were applied based on normality tests. The McNemar test was used for categorical variables. * Non-parametric variables. *p* < 0.05. *p*^1^ Comparison between final vs. baseline in the active placebo group (intragroup). *p*^2^ Comparison between final vs. baseline of the marine ω-3 group (intragroup). *p*^3^ Comparison between the groups (intergroup: Active placebo group vs. marine ω-3 group.

**Table 3:** Dietary variables in the study groups: intragroup and intergroup comparison.

	Active Placebo Group (n = 7)				Marine ω-3 Group (n = 7)				
Dietetics									
Variable	Baseline	Final	∆	*p* ^1^	Baseline	Final	∆	*p* ^2^	*p* ^3^
Energy (kcal)	2123 ± 607.1	2093.3 ± 844.2	−29.7 ± 237.1	0.53	2049 ± 571.4	1805 ± 332.3	−244 ± 239.1	0.45	0.836
Protein (g/day)	84.0 ± 40.0	109.0 ± 30.5	25.0 ± 9.5	0.26	100.0 ± 25.8	81.3 ± 8.0	−18.7 ± 17	0.14	0.445
Carbohydrates (g/day)	189.8 ± 53.4	205.1 ± 100.2	15.3 ± 46.8	0.63	211.6 ± 75.4	184.5 ± 40.0	−27.1 ± 35.4	0.66	0.562
Lipids (g/day)	102.1 ± 45.3	87.3 ± 37.9 *	−14.8 ± 7.4	0.62	85.8 ± 38.8	76.5 ± 22.4	−9.3 ± 16.4	0.48	0.366

The variables are expressed as mean ± standard deviation. Independent *t*-tests or Mann–Whitney U tests were applied. Baseline comparisons of the variables between groups showed no statistically significant differences at the start of the intervention. Independent *t*-tests or Mann–Whitney U tests were used depending on the normality of the variables. * Non-parametric variables: *p* > 0.05. *p*^1^: Final vs. baseline comparison in the active placebo group. *p*^2^: Final vs. baseline comparison in the marine ω-3 group. *p*^3^: Baseline comparison between groups.

**Table 4:** Global consumption of ω-6 and ω-3 PUFA types in the study groups; baseline and final intragroup and intergroup comparisons.

	Active Placebo Group (n = 7)					Marine ω-3 Group (n = 7)						
Global Consumption of ω-6 and ω-3 PUFA												
Variable	Baseline	1st Month	Final	∆	*p* ^1^	Baseline	1st Month	Final	∆	*p* ^2^	*p* ^3^	*p* ^4^
ω-3 PUFA (g/day)	1.32 ± 0.41	3.08 ± 0.4	3.39 ± 1.11	1.8 ± 1.7	**0.04**	1.61 ± 0.53	4.24 ± 0.6	5.05 ± 0.94	3.4 ± 1.3	**<0.01**	0.127	0.150
EPA (g/day)	0.04 ± 0.03	0.02 ± 0.008	0.08 ± 0.1	0.07 ± 0.10	0.13	0.04 ± 0.02	1.02 ± 0.2	1.0 ± 0.08	0.96 ± 0.07	**<0.01**	0.684	**0.004**
DHA (g/day)	0.036 ± 0.029	0.097 ± 0.12	0.38 ± 0.34	0.28 ± 0.34	0.09	0.14 ± 0.16	0.84 ± 0.22	0.77 ± 0.12	0.63 ± 0.14	**<0.01**	0.081	0.055
ALA (g/day)	0.99 ± 0.39	3.9 ± 1.92	4.74 ± 1.75	3.0 ± 2.5	0.07	1.39 ± 0.52	2.29 ± 0.79	2.87 ± 1.32	1.4 ± 1.4	0.05	0.262	0.150
ω-6 PUFA (g/day)	14.6 ± 3.44	15.56 ± 5.7	18.96 ± 7.96	4.8 ± 9.1	0.30	15.5 ± 3.89	14.9 ± 2.33	15.14 ± 3.8	−0.36 ± 6.2	0.89	0.873	0.273
ω-6:ω-3 PUFA	16.2 ± 6.43	4.9 ± 1.33	5.88 ± 3.22	−10.5 ± 4.2	**<0.01**	11.27 ± 6.89	3.5 ± 0.77	3.08 ± 0.85	−8.1 ± 7.2	**0.04**	0.109	0.273

The variables are expressed as mean ± standard deviation. ω-3: omega-3, EPA: Eicosapentaenoic acid, DHA: Docosahexaenoic acid, ALA: Alpha-linolenic acid, ω-6: omega-6. Mann–Whitney U tests were applied. Baseline comparisons of the variables between groups showed no statistically significant differences at the start of the intervention. *p*^1^: Final vs. baseline comparison in the active placebo group. *p*^2^: Final vs. baseline comparison in the marine ω-3 group. *p*^3^: Baseline comparison between groups. *p*^4^: Comparison of changes (∆) between groups (intergroup).

**Table 5:** Overall adherence of both study groups (dietary ω-3 consumption and supplementation).

	Active Placebo Group (n = 7)				Marine ω-3 Group (n = 7)					
	1st Month	Final	∆	*p* ^1^	1st Month	Final	∆	*p* ^2^	*p* ^3^	*p* ^4^
Adherence (%)	68.0 ± 19.30%	66.6 ± 8.33%	−6.6 ± 12.3	0.235	79.1 ± 27.76%	69.4 ± 27.72%	−9.7 ± 21.3	0.285	0.440	0.851

The variables are expressed as mean ± standard deviation. Mann–Whitney U test was applied. *p*^1^: Final vs. baseline comparison in the active placebo group. *p*^2^: Final vs. baseline comparison in the marine ω-3 group. *p*^3^: Baseline comparison between groups. *p*^4^: Comparison of changes (∆) between groups (intergroup).

**Table 6:** Correlation between PUFA consumption and FFAR4 activation.

	Active Placebo Group (n = 7)		Marine ω-3 Group (n = 7)	
Variables	Correlation Coefficient	*p*	Correlation Coefficient	*p*
ω-3 PUFA (g/day)	0.200	0.70	0.086	0.87
EPA (g/day)	0.543	0.26	0.829	**0.04**
DHA (g/day)	0.543	0.26	0.600	0.20
LA (g/day)	0.200	0.70	−0.886	**0.01**
ω-6 PUFA (g/day)	0.200	0.74	−0.886	**0.01**
Ratio ω-6:ω-3 PUFA	−0.100	0.87	−0.486	0.32

ω-3: omega-3, EPA: Eicosapentaenoic Acid, DHA: Docosahexaenoic Acid, LA: Linoleic Acid, ω-6: omega-6.

**Table 7:** Correlation between FFAR4 activation and gene expression of inflammatory markers in the study population (complementary cohort n = 14).

Variable (Gene Expression)	Correlation Coefficient	*p*
*JNK* (final)	−0.737	**0.004**
*IKKB* (final)	0.138	0.669

*JNK*: Jun N-terminal Kinase; *IKKB*: Inhibitor of Nuclear Factor Kappa B Kinase Subunit beta.

**Table 8:** Analysis of serum inflammatory markers in the study groups.

	Active Placebo Group (n = 29)				Marine ω-3 Group (n = 26)					
Serum Inflammatory Markers										
	Baseline	Final	∆	*p* ^1^	Baseline	Final	∆	*p* ^2^	*p* ^3^	*p* ^4^
**TNF-α (pg/mL)**	0.57 ± 0.8	0.11 ± 0.09	−0.45 ± 0.2	**0.002**	0.87 ± 0.8	0.10 ± 0.1	−0.77 ± 0.8	**0.001**	0.471	0.570
**IL-6 (pg/mL)**	3.6 ± 3.2	4.0 ± 3.3	0.4 ± 2.0	0.363	4.0 ± 4.3	2.6 ± 2.7	−1.3 ± 2.6	**0.047**	0.792	**0.030**
**IL-18 (pg/mL)**	132.4 ± 35.8	105.4 ± 21.3	−26.9 ± 34.2	**0.002**	119.2 ± 27.6	92.0 ± 20.6	−27.2 ± 25.2	**0.001**	0.239	0.839
**IL-10 (pg/mL)**	6.4 ± 3.6	4.7 ± 3.7	−1.6 ± 3.4	0.088	5.98 ± 3.8	8.6 ± 7.5	2.3 ± 4.8	**0.024**	0.631	**0.004**

Variables expressed as mean and standard deviation. TNF-α: Tumor necrosis factor-alpha, IL-6: Interleukin 6, IL-18: Interleukin 18, IL-10: Interleukin 10. *p*^1^: Final vs. baseline comparison in the active placebo group. *p*^2^: Final vs. baseline comparison in the marine ω-3 group. *p*^3^: Baseline comparison between groups. *p*^4^*:* Comparison of differences (∆) between groups (intergroup).

**Table 9:** Correlation analysis of FFAR4 activation and serum inflammatory markers.

	Active Placebo Group (n = 7)		Marine ω-3 Group (n = 7)	
Variable	Correlation Coefficient	*p*	Correlation Coefficient	*p*
**TNF-α**	−0.750	0.052	−0.791	**0.034**
**IL-6**	0.348	0.444	−0.538	0.212
**IL-18**	−0.552	0.199	−0.631	0.128
**IL-10**	−0.393	0.383	0.930	**0.002**

TNF-α: Tumor necrosis factor-alpha, IL-6: Interleukin 6, IL-18: Interleukin 18, IL-10: Interleukin 10. Pearson/Spearman test according to normality. *p* < 0.05.

## Data Availability

The original contributions presented in this study are included in the article/Supplementary Material. Further inquiries can be directed to the corresponding authors.

## Supplementary Materials

The following supporting information can be downloaded at: https://www.mdpi.com/article/10.3390/nu17233630/s1, Figure S1: Immunoprecipitation and Western blot for acute consumption (positive control); Figure S2: Immunoprecipitation and Western blot for chronic consumption (positive control); Table S1: Treatment nutrition facts (Marine oil); Table S2: Active placebo nutrition facts (Flaxseed and chia oil); Table S3: Fatty acid profile of treatment (marine oil); Table S4: Fatty acid profile of active placebo (vegetable oil); Table S5: Demographic characteristics of the study groups at the beginning of the intervention.
