# Supplementary material for: Marine ω-3 PUFA Supplementation Enhances FFAR4 Activation and Reduces Inflammatory Markers in PBMC of Subjects with Obesity: A Randomized Controlled Trial (EPICO)
Source: Nutrients. 2025 Nov 21;17(23):3630. doi: 10.3390/nu17233630 (PMC12693359; doi:10.3390/nu17233630)
Supplement: Supplementary file 1 [file nutrients-17-03630-s001.zip › nutrients-3969245-supplementary.pdf]

Supplementary Material

Table S1. Treatment nutrition facts (Marine oil).

Oil source	Content of 1 capsule (1 g of fish oil)	
Mixed oil from: Anchovy, Mackerel, Salmon, Tuna, and Cod	EPA	360 mg
	DHA	240 mg
Total ω-3 PUFA	**600 mg**	

EPA: Eicosapentaenoic acid, DHA: Docosahexanoic acid.

Table S2. Active placebo nutrition facts (Flaxseed and chia oil).

Oil source	Content of 1 capsule (1 g of flaxseed and chia oil)	
Mixed oil from: Flaxseed oil and Chia seed	ALA	550 mg Total ω-3 PUFA

ALA: Alpha-linolenic acid.

Table S3. Fatty acid profile of treatment (marine oil).

Fatty acid	Percentage
14:0 (Myristic acid)	2.18
16:0 (Palmitic acid)	9.85
16:1 (Palmitoleic acid)	6.02
18:0 (Stearic acid)	0.94
18:1 (Oleic acid)	7.82
18:2 ω–6 (Linoleic acid)	1.09
18:3 ω–3 (Alpha-linolenic acid - ALA)	0.45
20:1 (Eicosenoic acid)	1.91
20:4 ω–6 (Arachidonic acid)	1.48
20:5 ω–3 (Eicosapentaenoic acid - EPA)	35.89
22:1 (Erucic acid)	0.96
22:5 ω–3 (Docosapentaenoic acid - DPA)	4.98
22:6 ω–3 (Docosahexaenoic acid - DHA)	23.91

Source: Mixed oil from anchovy, mackerel, salmon, tuna, and cod.

Table S4. Fatty acid profile of active placebo (vegetable oil)

Fatty acid	Percentage
16:0 (Palmitic acid)	4.87
18:0 (Stearic acid)	1.86
18:1 (Oleic acid)	10.94
18:2 ω–6 (Linoleic acid)	22.78
18:3 ω–3 (Alpha-linolenic acid - ALA)	55.53
20:0 (Arachidic acid)	1.04
22:0 (Behenic acid)	0.49

Source: Mixed oil from flaxseed and chia seed.

To validate controls for the the FFAR4/β-arrestin-2 immunoprecipitation and western blot procedures, a pilot study was performed including both acute and chronic ω-3 PUFA supplementation as positive controls in healthy subjects. Two healthy volunteers (n=2) were recruited for the acute ω-3 intake test, consuming 5 g of marine ω-3 PUFA 8 hours before sample collection. Venous blood was drawn after an overnight fast (8–12 h), and PBMCs were isolated by density gradient centrifugation using Lymphoprep. Total protein (5–7×10^9/L) was extracted using three different lysis buffers (RIPA, modified RIPA [mRIPA], and TRIzol). Protein quantification was performed by the Bradford method, and 150 μg of lysate was subjected to immunoprecipitation with an anti-β-arrestin-2 antibody, followed by standard western blot for FFAR4 detection. As shown in ***figure S1***, FFAR4 was detected in protein extracts obtained with RIPA and mRIPA buffers, whereas no FFAR4 signal was observed in TRIzol-derived samples, in which only β-arrestin-2 was detected. These findings suggest that TRIzol components may disrupt protein–protein interactions, making RIPA and mRIPA more suitable for FFAR4 activation assays.

Subsequently, a chronic ω-3 PUFA intake test was conducted in five healthy volunteers (n=5) who had consumed marine ω-3 fatty acids (2 g/day of EPA + DHA) for at least 30 days. PBMCs were isolated and lysed using mRIPA buffer, and proteins were quantified and subjected to immunoprecipitation and western blot as described above. As shown in ***figure S2***, both FFAR4 and β-arrestin-2 were consistently detected in these samples, confirming receptor activation after chronic ω-3 supplementation

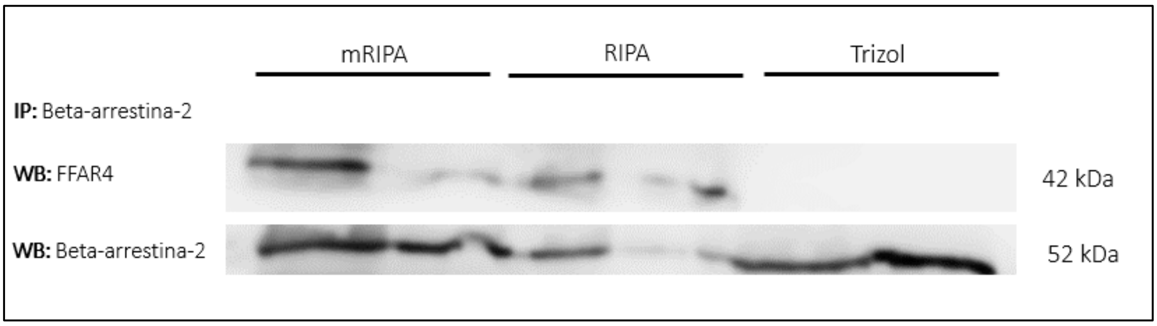

Figure S1. **Immunoprecipitation and Western blot for acute consumption (positive control).** Peripheral blood mononuclear cells (PBMCs) were isolated from peripheral blood samples (n = 2) obtained from healthy volunteers who had undergone acute supplementation with marine ω-3 fatty

acids (5 g, the night before evaluation). Immunoprecipitation was performed using an anti–β-arrestin-2 antibody, followed by a standard Western blot assay. FFAR4 and β-arrestin-2 were detected on the membrane.

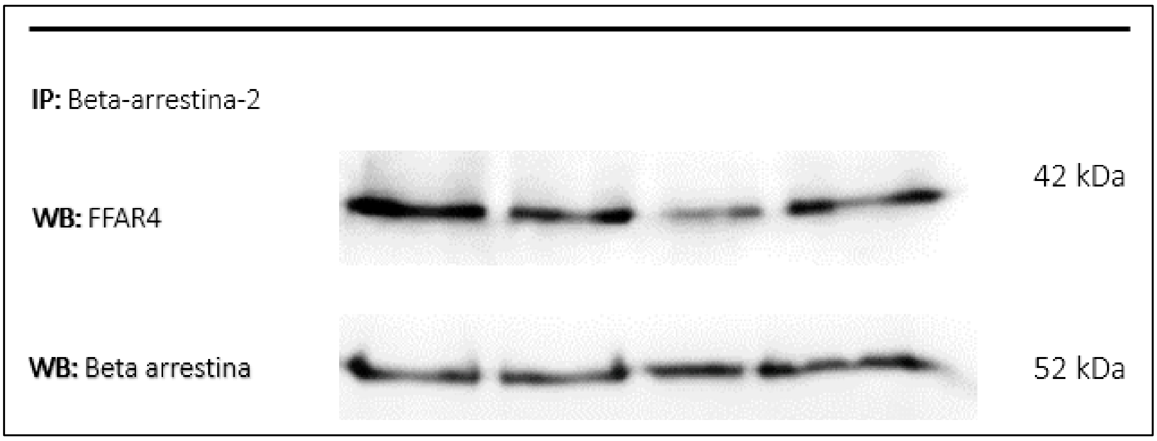

Figure S2. Immunoprecipitation and Western blot for chronic consumption (positive control) Peripheral blood mononuclear cells (PBMCs) were isolated from peripheral blood samples (n = 4) obtained from volunteers with chronic supplementation of marine ω-3 fatty acids (2 g/day for at least 30 days). Immunoprecipitation was performed using an anti–β-arrestin-2 antibody, followed by a standard Western blot assay. FFAR4 and β-arrestin-2 were detected on the membrane.

Table S5. Demographic characteristics of the study groups at the beginning of the intervention

Variable	Active placebo group (n=29)	Marine ω-3 group (n=26)	*p*
Age (years)	38.1±10.1	38.2±9.8	0.94
Sex			
Female (%)	13 (44.8)	11 (42.3)	0.85
Male (%)	16 (55.2)	15 (57.7)	

Qualitative variables expressed in frequency and percentage, parametric quantitative variables expressed in mean and standard deviation. Student's T test / χ^2
